# A rare case of supernumerary fused and malrotated kidney

**DOI:** 10.1590/S1677-5538.IBJU.2015.0420

**Published:** 2017

**Authors:** Volkan Sen, Ibrahim Halil Bozkurt, Tarik Yonguc, Ozgu Aydogdu, Ismail Basmaci

**Affiliations:** 1 Izmir Bozyaka Training and Research Hospital – Urology, Izmir, Turkey

## MANUSCRIPT

The supernumerary kidney is an accessory organ with its own blood supply and collecting system. It is a very rare type of congenital renal anomaly with fewer than 100 cases reported since firstly described at 1656 (1). Embryological basis of supernumerary kidney is connected to the abnormal division of the nephrogenic cord into two metanephric blastemas which will form two kidneys (2). It may be either completely separate or only loosely attached to the major kidney on the ipsilateral side. This anomaly is usually asymptomatic but may rarely become symptomatic in early adulthood (3). The mean age at diagnosis is 36 years. The most common presenting symptoms are pain, fever and a palpable abdominal mass. Ultrasonography, CT and MR urography may be needed to identify the anomaly.

**Figure 1:**
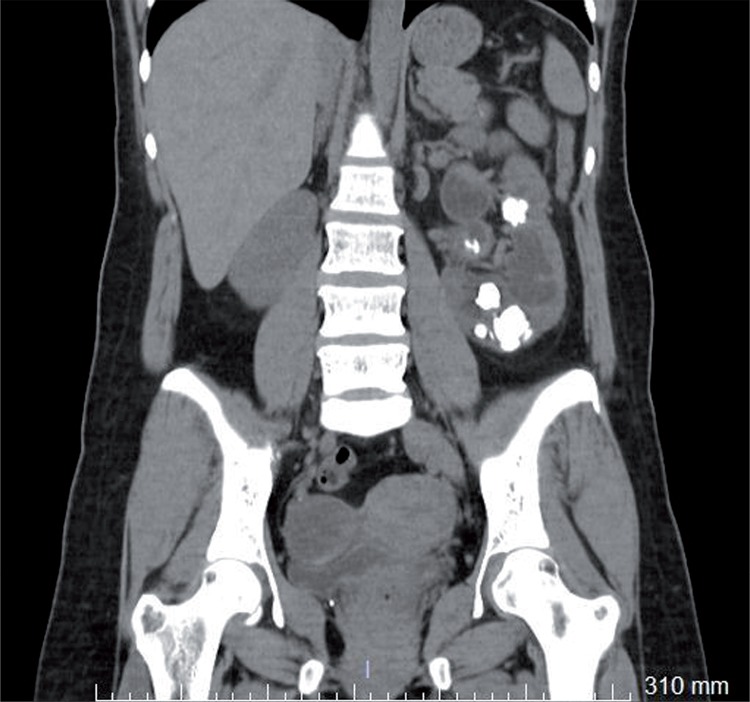
Computed tomography scan with multiple calculus in left kidney.

**Figure 2:**
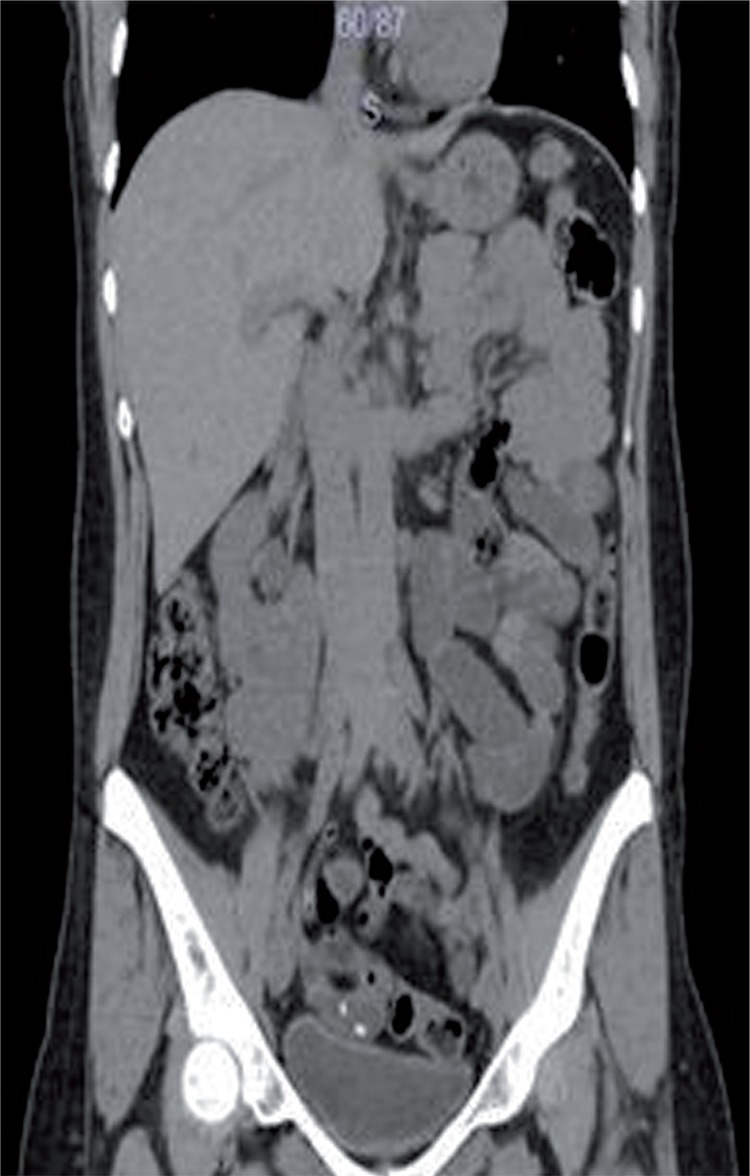
Computed tomography scan of right kidney.

**Figure 3:**
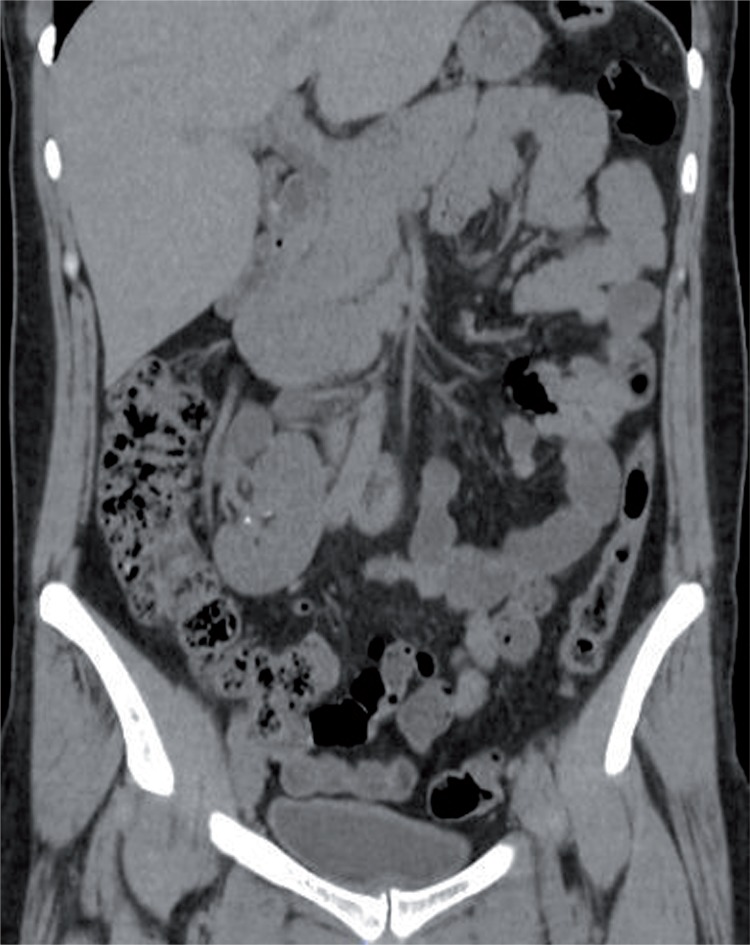
Computed tomography scan of supernumerary kidney with its own blood supply and collecting system.

We aimed to present a rare case of supernumerary fused and malrotated kidney. Thirty-seven year-old woman was admitted to our clinic with left flank pain. CT scan demonstrated multiple calculi in the left kidney and supernumerary fused kidney (Figures 1 and 2). The third kidney was below the right kidney, malrotated and had its own blood supply (Figure-3).

Grieshammer and colleagues showed that mutant mice lacking either SLIT2 or its receptor ROBO2 develop supernumerary ureteric buds that are correlated with abnormal maintenance of Gdnf expression in anterior metanephric mesenchyme (4). The SLIT2/ROBO2 intercellular signaling system restricts, directly or indirectly, in extent of the Gdnf expression and plays a critical role in precisely positioning the side of kidney induction. The rare location and malrotation of supernumerary kidney of our case could be explained by this hypothesis. Percutaneous nephrolithotomy was performed to the left kidney and no problem was observed in follow-up.

## References

[1] Sureka B, Mittal MK, Mittal A, Sinha M, Thukral BB (2014). Supernumerary kidneys—a rare anatomic variant. Surg Radiol Anat.

[2] Tada Y, Kokado Y, Hashinaka Y, Kadowaki T, Takasugi Y, Shin T (1981). Free supernumerary kidney: a case report and review. J Urol.

[3] CARLSON HE (1950). Supernumerary kidney: a summary of 51 reported cases. J Urol.

[4] Grieshammer U, Ma Le, Plump AS, Wang F, Tessier-Lavigne M, Martin GR (2004). SLIT2-mediated ROBO2 signaling restricts kidney induction to a single site. Dev Cell.

